# Stoichiometric Ratio Controlled Dimension Transition and Supramolecular Chirality Enhancement in a Two-Component Assembly System

**DOI:** 10.3390/gels8050269

**Published:** 2022-04-26

**Authors:** Penghui Zhang, Yiran Liu, Xinkuo Fang, Li Ma, Yuanyuan Wang, Lukang Ji

**Affiliations:** 1 Hebei Key Laboratory of Organic Functional Molecules, College of Chemistry and Materials Science, Hebei Normal University, Shijiazhuang 050024, China; 17599090428@stu.hebtu.edu.cn (P.Z.); liuyiran@stu.hebtu.edu.cn (Y.L.); xinkuo86@163.com (X.F.); 2 Department of Pharmacology, College of Basic Medicine, Hebei University of Chinese Medicine, Shijiazhuang 050200, China

**Keywords:** stoichiometric ratio, dimension transition, supramolecular chirality, two-component gel

## Abstract

To control the dimension of the supramolecular system was of great significance. We construct a two component self-assembly system, in which the gelator LHC18 and achiral azobenzene carboxylic acid could co-assembly and form gels. By modulating the stoichiometric ratio of the two components, not only the morphology could be transformed from 1D nanaotube to 0D nanospheres but also the supramolecualr chirality could be tuned. This work could provide some insights to the control of dimension and the supramolecular chirality in the two-component systems by simply modulating the stoichiometric ratio.

## 1. Introduction

The control of dimension is crucial in nanomaterials and bioscience [1,2,3]. As the dimension are varied, the properties and functions of the nanomaterials will dramatically change [4,5,6]. For instance, in carbon nanomaterial aspects, 0D fullerenes, 1D nanotubes, 2D graphene, and 3D graphite had different dimensions and thus lead to distinct electronic and photonic properties (in which 0D, 1D, 2D, 3D represents zero, one, two, and three dimension, respectively). Similarly, in biosciences, proteins form nanostructures including 1D microtubules, 2D bacterial surface layers, and 3D virus capsids with distinct structure and functions, i.e., the dimension information not only had tight relationships with the corresponding functional expression but also had great influence on the pathological changes or diseases in bioscience [7,8,9]. Supramolecular systems are formed by the weak non-covalent interactions including hydrogen bond, pi-pi stacking and hydrophobic effect [10,11,12,13,14,15]. By the synergy of one or multi-interactions, nanostructures such as 0D nanospheres, 1D nanotubes and 2D nanoplates could form. Moreover, the dimension of the supramolecular assemblies played a crucial role in the applications, such as the 1D gold nanorod had photothermal effect and had been used in the tumor therapy fields, however the effect cannot be realized by 0D gold nanoparticle [16]. Thus, it is significant to explore the relationships between the dimension transition and functions in supramolecular assemblies.

Benefit by the dynamic properties of the supramolecular systems, many approaches could be utilized to modulate the dimension of the assemblies. For examples, Zhao and co-workers reported that by light irradiation, the morphology of the azobenzene derivative assemblies transform from 2D nanosheet to 1D nanotube and 0D nanoparticles by the *Z*-*E* isomerization of the azobenzene moiety [17]. Kawai and co-workers reported that the dimension of the assemblies could be tuned by solvents, and the *g* factor of circularly polarized luminescence can be promoted significantly [18]. Therefore it could be concluded that not only the supramolecular structures but also the dimension of the assembly could affect the intensity of the chirality and even the direction of the helicity [19,20,21,22,23,24,25,26].

Two-component self-assembly systems referring to two kinds of materials were involved in the assembly process, such as two types of organic molecule or organic molecule with inorganic nanoparticles et al. [27,28,29,30,31,32]. In two-component self-assembly systems, new properties or functions could appear, thus inducing many superiorities compared with the traditional one-component systems. For examples, in cyclodextrin, calixarenes, and chiral host-based host-guest systems, by the co-assembly process the chirality could be transferred from the chiral host to the achiral guests [33,34,35,36,37,38,39,40,41,42,43]. In the light harvesting systems, by doping the energy acceptor to the chiral donor assemblies, not only the fluorescent emission wavelength could be changed but also the utilization effect of the light could be promoted [44,45,46]. Moreover, by co-assembly strategy, the supramolecular chirality of the systems could be modulated, such as enhancement and inversion of the chirality or the helicity of the nanostructures [47,48,49,50,51,52,53].

In the two-component self-assembly systems, stoichiometric ratio of the components could be used to tune the supramolecular chirality and the nanostructure of the assemblies. For instance, Liu and co-workers control both the length and the diameter of the nanotubes only by the ratio of the gelator and melamine [54]. Yin and co-workers realized the inversion of the CPL signals by modulating the stoichiometric ratio of the chiral gelator and the achiral AIEgen lumiphores [55]. However, by regulating the stoichiometric ratio of the two-component in the co-assembly systems to realize both the dimension transition and chirality intensity control was rarely reported.

Herein, we construct a two-component supramolecualr system including a chaperone gelator LHC18 and a bola-type azobenzene carboxylic acid. Gelator LHC18 is an amphiphilic L-histidine derivative contains a long alkyl chain and a urea bond, LHC18 can self-assemble to supramolecular nanotwist in the mixed solution of DMF and H_2_O by hydrogen bond and hydrophobic effect [56]. Moreover, LHC18 could form two-component gel with azobenzene derivatives by the interaction between the imidazole and carboxylic acid group and exhibit distinct morphology and chiroptical properties. By modulating the stoichiometric ratio of the two components, we will explore that weather the two-component assembly could show dimension transformation and chirality enhancement.

## 2. Results and Discussion

**Figure 1:**
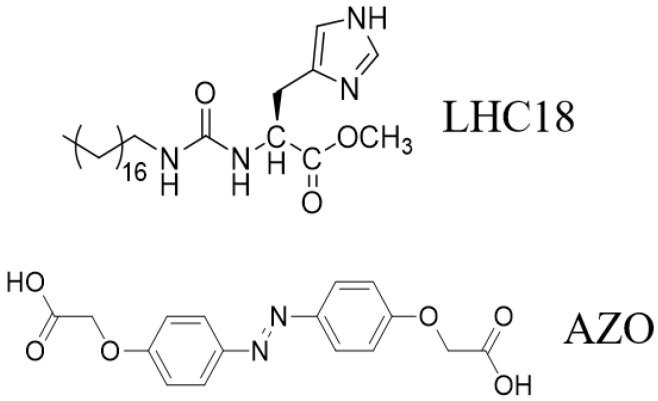
Molecular structure of chiral gelator LHC18 and achiral AZO.

**Figure 2:**
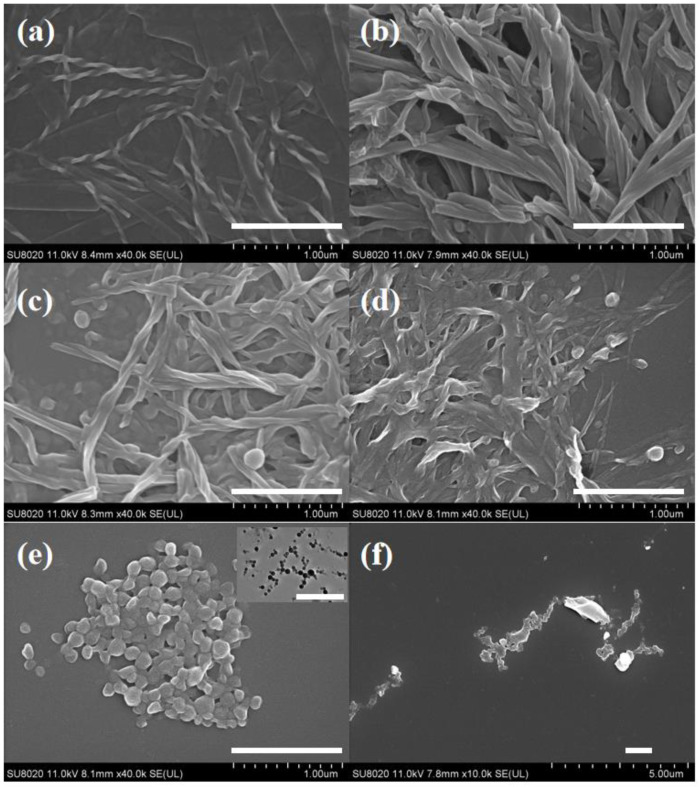
SEM images of (**a**) LHC18 assembly (DMF/H_2_O = 500 μL/900 μL), and LHC18/AZO assemblies (DMF/H_2_O = 300 μL/600 μL) at different molar ratios: (**b**) 1/0.5, (**c**) 1/1, (**d**) 1/2, (**e**) 1/4. (**f**) AZO precipitate (DMF/H_2_O = 300 μL/600 μL). The inserted image was the TEM image of the assembly of 1/4. Scale bar 1 μm.

Carboxylic acid-terminated achiral azobenzene derivatives AZO as shown in Figure 1, could form hetero-hydrogen bond with imidazole moiety of LHC18, while the ratio of AZO was exceeded, AZO itself could form homo-hydrogen bond. Therefore, we suppose that by modulating the stoichiometric ratio of the two components will change the stacking mode thus affect the chirality transfer from LHC18 to AZO and induce the morphology and even dimension transition. LHC18 itself can dissolve in DMF solution, to obtain the supramolecular gel anti-solvent method was applied. 5 mg LHC18 was dissolved and heat in 500 DMF μL, the the boiled Milli-Q water was injected, after about ten minutes an opaque white supramolecular gel was formed proved by inverted tests (Figure S1) and rheological data (Figure S2), and nanotwist structure can be measured as shown in Figure 2a, the enlarged images is listed in Figure 2.

For the formation process of the co-assemblies, 5 mg LHC18 and various ratios of AZO were added into 300 μL DMF. The mixture was heated to form a transparent solution and then 600 μL boiled Milli-Q water was injected, after about 10 min supramolecular could be obtained. AZO (3.45 mg) itself cannot form ordered nanostructures in the mixed solution, it only forms some precipitates as showed in the SEM image (Figure 2f).

Next, we modulated the stoichiometric ratio of LHC18 and AZO to explore the morphology change. When LHC18 co-assembled with AZO at a molar ratio of 1/0.5, i.e., the molar ratio of the imidazole and the carboxylic acid group was 1/1, an opaque orange gel can be obtained, which was confirmed by inverted test tube experiments (Figure S1) and rheological data (Figure S3), nanotube can be observed by the SEM measurement and further proved by TEM image (Figure S4).

When the proportion of AZO was increased, some new phenomena could be found, at the molar ratio of LHC18/AZO was changed to 1/1 and 1/2, nanofibers and spheres can be observed from the SEM images (Figure 2c,d). However, as the increasing of AZO to the radio 1/4, only spheres can be found in the SEM images and the spheres are solid as proved by the TEM image, Figure 2e top right corner. It should be noted that the nanotwist of pure LHC18 is left-handed (*M*), after adding AZO to the ratio is 1/0.5, the supramolecular chirality of nanostructure was inverted to right-handed (*P*). Moreover, by observing the reagent bottle after co-assembly process, only the assembly of the ratio was 1/0.5 could form gel, the assemblies of other ratios only had the sol phase, Figure S1. The above results indicated that by modulating the stoichiometric ratio of LHC18 and AZO, not only the morphology but also the dimension could be altered.

**Figure 3:**
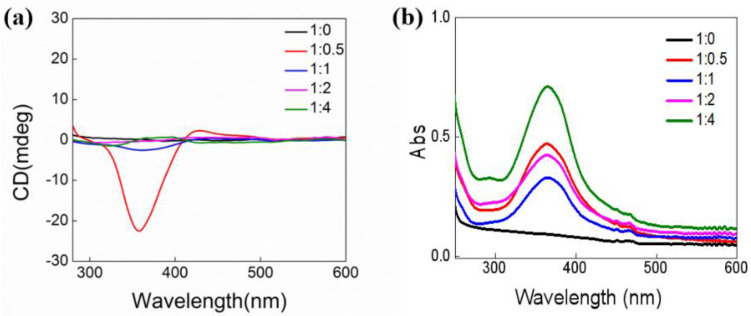
(**a**) CD and (**b**) UV-vis spectrum of LHC18/AZO at different molar radio, LHC18 was kept constant at 5 mg in the co-assemblies.

For LHC18 was a chiral molecule, we also test the chirality transfer from LHC18 to AZO at different molar ratio. As shown in Figure 3, for LHC18 had no obvious Uv-vis absorption at 300–400 nm, the CD spectra had no signals. However, for the co-assembly of LHC18/AZO at molar ratio was 1/0.5, evident CD signals could be observed. The CD spectra showed a positive cotton effect with bisignated splitting. The positive and negative cotton effect centered at 424 nm and 354 nm with a crossover at 401 nm. The *trans*-isomer of AZO had absorption at 364 nm which could be ascribed to the π–π* absorption band, so the excited couplings of the AZO chromophore were responsible for the CD signals. Moreover, the CD spectra showed a positive cotton effect thus indicating that the AZO moiety adopted a *P*-helicity stacking mode, i.e., the azobenzene chromophore stacked in a clockwise direction [57,58].

**Figure 4:**
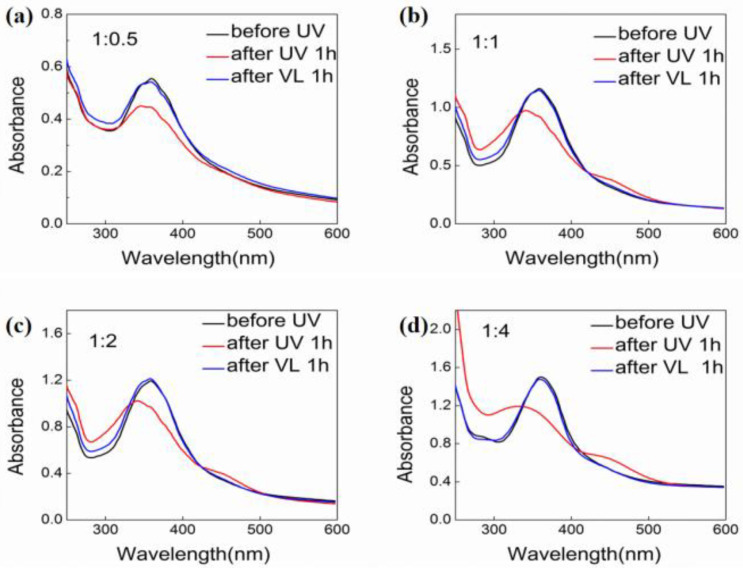
Uv-vis spectra of LHC18/AZO assemblies after UV 365 nm irradiation for one hour at different molar radio, (**a**) 1/0.5, (**b**) 1/1, (**c**) 1/2, and (**d**) 1/4.

However, as the molar ratio of the AZO increased, the CD signals was distinctly decreased. As showed in Figure 3, only the co-assembly with molar ratio 1/1 had a weak signal, the co-assemblies of 1/2 and 1/4 nearly kept silence in the CD spectra. The CD tests suggested that only the stoichiometric ratio kept at a suitable value the chirality transfer can be effectively realized. For azobenzene moiety was light responsive which can undergo *trans*-*cis* isomerization, we also tested the chiroptical properties. As shown in Figure 4, the Uv-vis spectra showed that after UV 365 nm irradiation for 1 h, all the co-assembly spectra had obvious changes, after UV irradiation for one hours the assembly with molar ratio 1/0.5 had only intensity change indicating that the *trans*-*cis* isomerization was hampered, one AZO molecule was fixed by two LHC18 molecule thus limiting the isomerization. As the amount of AZO increased, AZO could have more flexibility thus the isomerizaiton could proceed. For the assemblies with the molar ratio was 1/1, 1/2 and 1/4, the 364 nm absorption band attributed to the π–π* of the *trans*-isomer decreased with the emergence of the peak at 310 nm and 440 nm corresponding to the π–π* and n–π* bands of the *cis*-isomer, respectively. After visible light irradiation for one hour, the 364 nm absorption band of the *trans*-isomer appeared again accompanied with the vanishment of the 310 nm and 440 nm signals of the *cis*-isomer. The UV-Vis result demonstrated the azobenzene *trans*-*cis* transition could be triggered by UV or visible light at the molar ratio was 1/1, 1/2, and 1/4.

**Figure 5:**
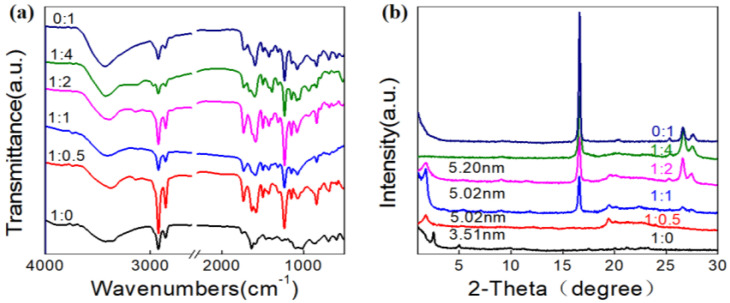
FT-IR spectra (**a**) and XRD patterns (**b**) of LHC18/AZO assemblies with different molar ratios.

In order to deeply understand the intermolecular interactions, we further investigated the FT-IR spectra and XRD patterns. All the samples are prepared from either the xerogel or the air-dried suspensions. The FT-IR spectra showed some common characteristics. Firstly, there are wide vibration bands around 1950 cm^−1^ for all the LHC18/AZO samples, which can be attributed to the formation of the hydrogen bond between imidazole and carboxylic acid group. Secondly, all the FT-IR spectra showed strong vibrations at around 1620 cm^−1^, which demonstrated a strong and ordered hydrogen bonding between the urea groups. Finally, all the samples of the asymmetric and symmetric stretching vibrations of CH_2_ appear at about 2920 cm^−1^ and 2850 cm^−1^, respectively, indicating that the alkyl chains are in the existence of considerable gauche conformations [59,60,61,62,63]. The XRD patterns of all the samples are also shown in Figure 5. Based on the Bragg’s equation, the *d*-spacing value of the samples can be estimated to be 3.51 nm, 5.02 nm and 5.20 nm. Since the distance 3.51 nm is larger than one but much less than twice the molecular length of the LHC18, we indicate that LHC18 forms an interdigitated bilayer structure. When the molar ratio of LHC18/AZO is 1/0.5, 1/1, and 1/2, the distance 5.02 nm and 5.20 nm is larger than one but slightly less than twice the molecular length of the LHC18, we suppose that it was the *d*-value of bilayer LHC18 and one AZO molecule. For the XRD patterns of LHC18/AZO is 1/1, 1/2, 1/4 and 0/1 systems, scattering peak intensity at 2θ = 16.6 (0.53 nm) gradually increased, indicating that the proportion of AZO which did not participate the co-assembly was improved. Moreover, the intensity peaks at 2θ = 26.62 and 27.46 (0.33 nm and 0.32 nm) also enhanced as the molar ratio of AZO increased, suggesting that the π–π stackings among the azobenzene groups appeared and increased.

**Figure 6:**
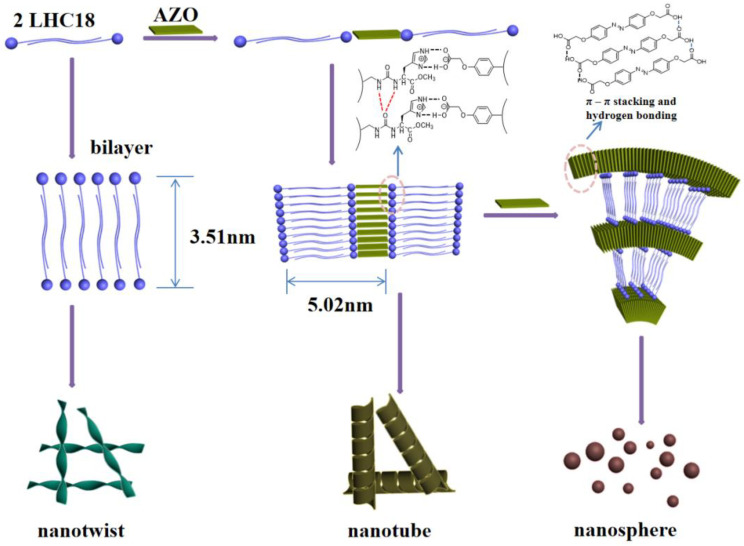
Illustration of the LHC18 and AZO co-assembly process. As the molar ratio of AZO increased, the chirality transfer was weakened, and the dimension transited from 1D nanotube to 0D nanosphere.

Based on the above mentioned results, a possible mechanism to explain the dimension transition and chirality transfer is shown in Figure 6. Firstly, LHC18 itself formed bilayer structure and self-assembled into nanotwists, the hydrophobic effect and the hydrogen bonding between the urea groups is the main driving force to form bilayer structure. When LHC18 co-assembly with AZO with the ratio is 1/0.5, nanotubes could be formed. Hydrogen bond could be formed between imidazole groups of LHC18 and carboxylic acid groups of the AZO, which is verified by the FT-IR spectra. When the molar ratio is higher than 1/0.5, AZO has tendency to form π–π stacking and hydrogen bond among the carboxylic acid groups, thus influence the stacking mode and leading to the nanostructure change. When the ratio is 1/4, the π–π stacking at 0.33 nm and 0.32 nm dramatically enhanced and form nanopheres. Therefore, the π–π stacking and hydrogen bond between AZO synergistically induce the morphology and chirality change.

## 3. Conclusions

In conclusion, we construct the two-component self-assembly system. By modulating the stoichiometric ratio of the components of chaperone gelator LHC18 and AZO derivatives, both the dimension transition and chirality transfer could be modulated. It was proved that the π–π stacking and hydrogen bond among AZO was the main reason for the assembly dimension transition and chirality transfer altering. We believe our work will provide some insights and approach into the dimension and chirality control in the self-assembly systems.

## 4. Materials and Methods

All the starting materials and solvents were obtained from commercial suppliers and used as received. The synthesis route and the characterization were in reference [60] A typical procedure for the assembly formation in mixed solution was as follows: 5.0 mg of LHC18 and 1.8 mg AZO were mixed in 300 μL DMF solution, heat to form a transparent solution, and then injected 600 μL boiled Milli-Q water, the mix solution was cooled to room temperature naturally. UV−Vis spectra were recorded in quartz curvettes (light path 0.1 mm) on a JASCO UV-550 spectrometer.

Circular Dichroism spectra were recorded in quartz cuvettes (light path 0.1 mm) on a JASCO J-810 spectrophotometer. The samples were prepared and cast on quartz plates. FT-IR spectra were recorded on a Bruker Tensor 27 FTIR spectrometer at room temperature. The KBr pellets made from the vacuum-dried samples were used for FT-IR spectra measurements. Scanning Electron Microscopy were cast onto single-crystal silica plates, the solvent was evaporated under the ambient conditions, and then vacuum-dried. The sample surface was coated with a thin layer of Pt to increase the contrast. SEM images were recorded on a Hitachi SU-8020 SEM instrument with an accelerating voltage of 11 kV. Transmission electron microscopy images were obtained on a JEM-1011 electron microscope at an accelerating voltage of 100 kV. The TEM samples were prepared by casting a small amount of sample on carbon-coated copper grids (300 mesh) and dried under strong vacuum. X-ray Diffraction (XRD) analysis was performed on a Rigaku D/Max-2500 X-ray diffract meter (Japan) with Cu Kα radiation (λ = 1.5406 Å), which was operated at a voltage of 40 kV and a current of 200 mA. Samples were cast on glass substrates and vacuum-dried for XRD measurements. Rheology Study were measured on a strain-controlled rheometer (MC-RhR 302, Anton Paar) using cone-plate geometry (25 mm diameter). The experiments were performed at 25 ± 0.05 °C, and the temperature was controlled with an integrated electrical heater.

## Data Availability

Not applicable.

## Supplementary Materials

The following supporting information can be downloaded at: https://www.mdpi.com/article/10.3390/gels8050269/s1, Figure S1: Phase image of the LHC18/AZO assemblies with different molar ratios. Figure S2: Rheological data for the LHC18 gel [64]. Figure S3: Rheological data for the LHC18/AZO gel. Figure S4: TEM image of the assembly of LHC18/AZO. Figure S5: CD spectra of the gel LHC18/AZO (molar ratio 1/0.5) with rotating the cuvette 90°. Figure S6: The SEM image of the LHC18/AZO assemblies under UV irradiation.
