# Supplementary material for: Stoichiometric Ratio Controlled Dimension Transition and Supramolecular Chirality Enhancement in a Two-Component Assembly System
Source: Gels. 2022 Apr 26;8(5):269. doi: 10.3390/gels8050269 (PMC9140661; doi:10.3390/gels8050269)
Supplement: Supplementary file 1 [file gels-08-00269-s001.zip › gels-1688193-supplementary.pdf]

gels

Supplementary Materials

Stoichiometric Ratio Controlled Dimension Transition and Supramolecular Chirality Enhancement in a Two-Component Assembly System

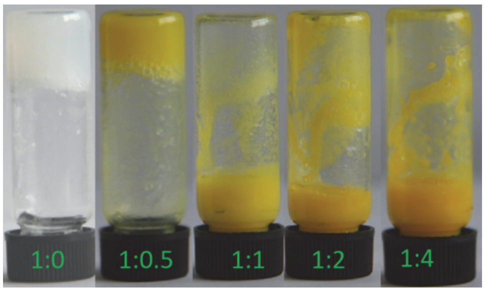

Figure S1. Phase image of the LHC18/AZO assemblies with different molar ratios.

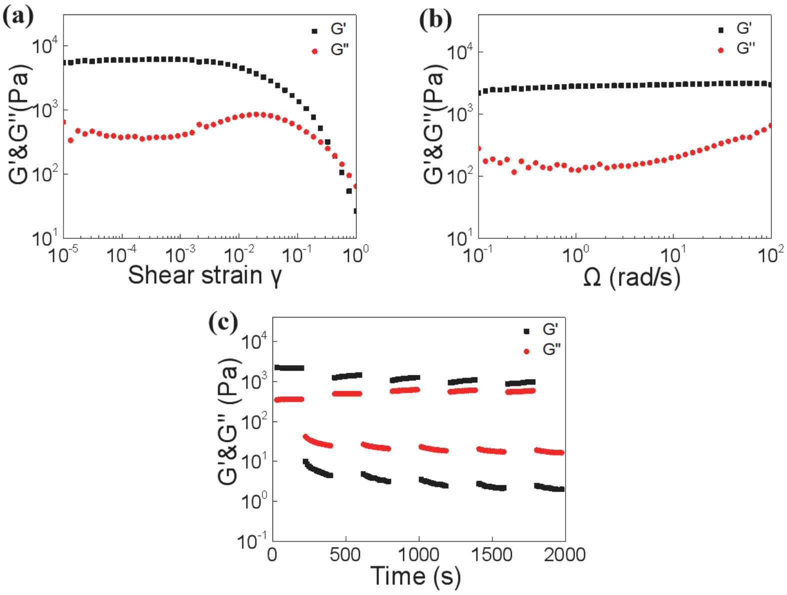

Figure S2. (**a**) Oscillatory strain sweep (1 rad s^{-1}, 0.001% - 100%), (**b**) Dynamic frequency sweep (0.1-100 rad s^{-1}, strain 0.01%), (**c**) Time-resolved sweep experiments (strain = 1% or 100%, 6.28 rad s^{-1}) for LHC18 gel in DMF/H_2O.

Rheological analysis was conducted to characterize the supramolecular gel. The experiment was conducted at the concentration of LHC18 7.70 mM and LHC18/AZO (2/1) 11.97 mM. For the dynamic sweep, when the applied strain exceeded the critical strain value 0.28% (LHC18) and 0.12% (LHC18/AZO), both G′ and G″ reduced dramatically, which implied the breakage of the cross-linked network under large strain. Frequency sweep data showed that the storage modulus G′ was larger than the loss modulus G′′, and the G′ was almost independent of the ω (angular frequency) for both LHC18 and LHC18/AZO assemblies, indicating the quasi-solid nature of the supramolecular gel. Cyclic strain sweep experiments were also performed to investigate the self-healing ability of the supramolecular gel. When a 100% strain was applied, the G′ and G″ values reduced dramatically and G″ was higher than G′, indicating the collapse of the supramolecular gel. However, when a 1% strain was applied, G′ and G″ nearly recovered to initial values, suggesting the supramolecular gel recovered, this process was reversible and

could be reproducible for multiple cycles. Rheological experiments indicated that the supramolecular gel had outstanding self-healing capability [1].

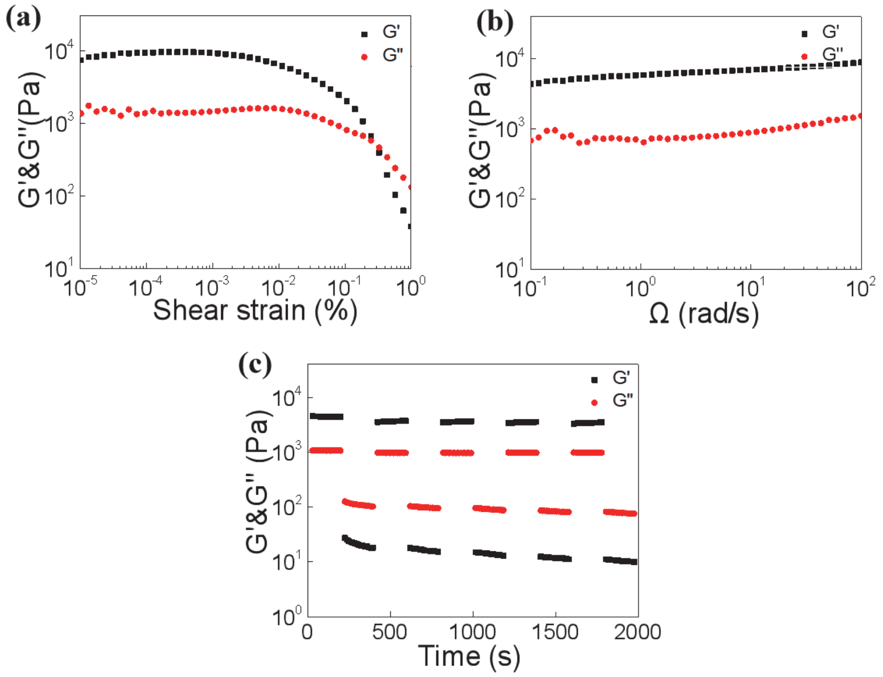

Figure S3. (**a**) Oscillatory strain sweep (1 rad s^{-1}, 0.001% - 100%), (**b**) Dynamic frequency sweep (0.1-100 rad s^{-1}, strain 0.01%), (**c**) Time-resolved sweep experiments (strain = 1% or 100%, 6.28 rad s $^{-1}$) for LHC18/AZO (molar ratio = 1/0.5) gel in DMF/H_2O.

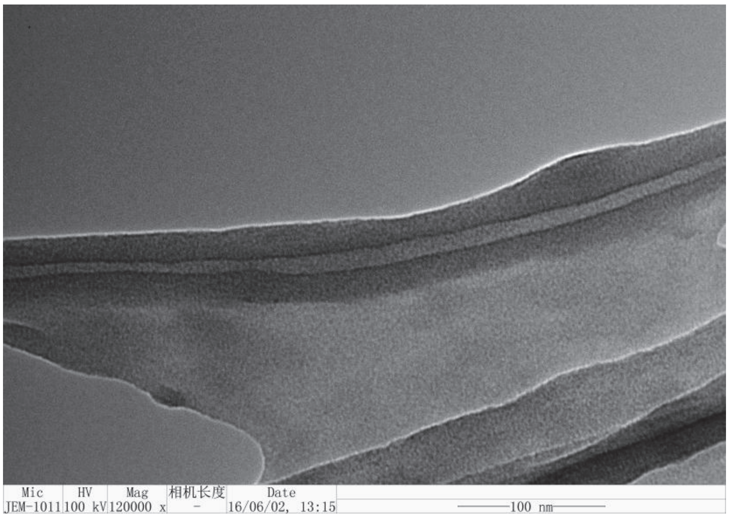

Figure S4. TEM image of the assembly of LHC18/AZO at the molar ratio was 1/0.5, it showed a nanotube structure.

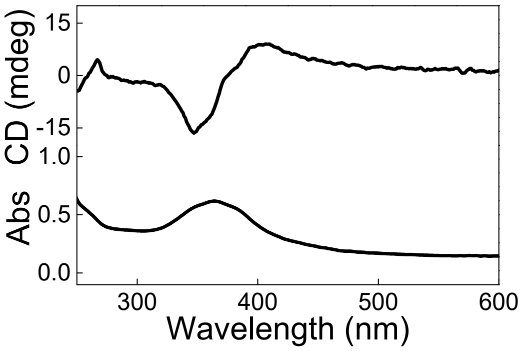

Figure S5. CD spectra of the gel LHC18/AZO (molar ratio 1/0.5) with rotating the cuvette 90°.

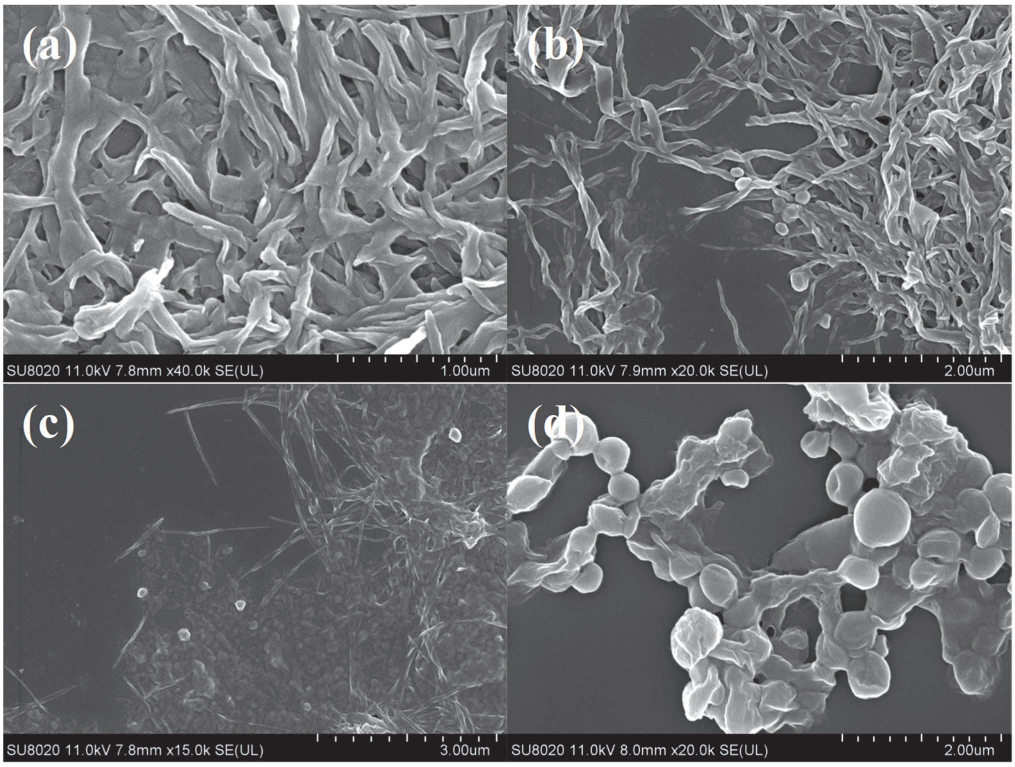

Figure S6. The SEM image of the LHC18/AZO assemblies with different molar ratios under UV irradiation for one hours, (**a**) 1/0.5, (**b**)1/1, (**c**)1/2, (**d**)1/4.

Reference

1. Li, H.; Duan, Z.; Yang, Y.; Xu, F.; Chen, M.; Liang, T.; Bai, Y.; Li, R., Regulable Aggregation-Induced Emission Supramolecular Polymer and Gel Based on Self-sorting Assembly. *Macromolecules* **2020**, 53, 4255-4263.
